# Characterisation of Deubiquitylating Enzymes in the Cellular Response to High-LET Ionizing Radiation and Complex DNA Damage

**DOI:** 10.1016/j.ijrobp.2019.02.053

**Published:** 2019-07-01

**Authors:** Rachel J. Carter, Catherine M. Nickson, James M. Thompson, Andrzej Kacperek, Mark A. Hill, Jason L. Parsons

**Affiliations:** ∗ Cancer Research Centre, Department of Molecular and Clinical Cancer Medicine, University of Liverpool, Liverpool, United Kingdom; † CRUK/MRC Oxford Institute for Radiation Oncology, University of Oxford, Gray Laboratories, Oxford, United Kingdom; ‡ The National Eye Proton Therapy Centre, The Clatterbridge Cancer Centre NHS Foundation Trust, Bebington, United Kingdom

## Abstract

**Purpose:**

Ionizing radiation, particular high-linear energy transfer (LET) radiation, can induce complex DNA damage (CDD) wherein 2 or more DNA lesions are induced in close proximity, which contributes significantly to the cell killing effects. However, knowledge of the enzymes and mechanisms involved in coordinating the recognition and processing of CDD in cellular DNA are currently lacking.

**Methods and Materials:**

A small interfering RNA screen of deubiquitylation enzymes was conducted in HeLa cells irradiated with high-LET α-particles or protons, versus low-LET protons and x-rays, and cell survival was monitored by clonogenic assays. Candidates whose depletion led to decreased cell survival specifically in response to high-LET radiation were validated in both HeLa and oropharyngeal squamous cell carcinoma (UMSCC74A) cells, and the association with CDD repair was confirmed using an enzyme modified neutral comet assay.

**Results:**

Depletion of USP6 decreased cell survival specifically after high-LET α-particles and protons, but not low-LET protons or x-rays. USP6 depletion caused cell cycle arrest and a deficiency in CDD repair mediated through instability of poly(ADP-ribose) polymerase-1 (PARP-1) protein. Increased radiosensitivity of cells to high-LET protons as a consequence of defective CDD repair was furthermore mimicked using the PARP inhibitor olaparib, and through PARP-1 small interfering RNA.

**Conclusions:**

USP6 controls cell survival in response to high-LET radiation by stabilizing PARP-1 protein levels, which is essential for CDD repair. We also describe synergy between CDD induced by high-LET protons and PARP inhibition, or PARP-1 depletion, in effective cancer cell killing.

**Summary**

Complex DNA damage (CDD) formation, which increases with increasing linear energy transfer, is a major contributor to the therapeutic effect of radiation therapy. However, little is known of the enzymes and mechanisms that control the cellular response to CDD and coordinate its repair. Using small interfering RNA screening of deubiquitylating enzymes, we identify major roles for USP6 and ultimately PARP-1 protein in regulating CDD repair and promoting cell survival in response to high linear energy transfer radiation.

## Introduction

DNA is the critical cellular target for ionizing radiation (IR), and the induction of DNA double-strand breaks (DSBs) and complex (clustered) DNA damage (CDD) is thought to be critical in contributing to the cell killing effects of IR.^1^ CDD is recognized as 2 or more DNA lesions induced in close proximity (eg, within 1-2 helical turns of the DNA) and has been demonstrated to persist in cells and tissues several hours post-IR as a result of the difficulty in their repair.^2^, ^3^ CDD formation increases with increasing linear energy transfer (LET) and has been predicted by mathematical modelling to be an important factor after proton beam irradiation, particularly at or around the Bragg peak, where low-energy protons (with increased LET) are generated.^4^, ^5^, ^6^ This has been shown indirectly by demonstrating that protons with increasing LET cause reductions in cell survival^7^, ^8^ and increases in persistent DNA DSBs as revealed by 53BP1 foci.^9^ However, recent data from our laboratory has directly demonstrated using an enzyme-modified neutral comet assay that low-energy (relatively high-LET) protons generate significantly increased amounts of CDD compared to high-energy (low-LET) protons or x-rays, which persists for several hours after irradiation.^10^

Given that CDD is known to be important in the cell killing effects of IR, the molecular and cellular mechanisms that respond to CDD within cellular DNA have been understudied. However, we recently demonstrated that CDD induced by high-LET protons and α-particles causes elevations in the levels of histone H2B ubiquitylation on lysine 120 (H2B_ub_). We discovered that this is coordinated by the E3 ubiquitin ligases RNF20/40 and MSL2, which play important roles in the repair of CDD and in cell survival after high-LET protons. We postulated that this is a mechanism for enhancing CDD repair by promoting chromatin remodeling or actively recruiting DNA repair enzymes.^10^ Nevertheless, this study found that ubiquitylation, particularly of histones, plays an important role in the cellular response to IR-induced CDD. Other DNA repair pathways, particularly DSB repair, are also known to be actively controlled by histone ubiquitylation that enhances DNA damage accessibility.^11^

In addition to regulation of DNA repair via controlling chromatin accessibility, numerous studies have demonstrated that DNA repair proteins themselves are subject to regulation by ubiquitylation, including those involved in DSB repair and in the repair of DNA base damage through the base excision repair pathway.^11^, ^12^, ^13^ This can be achieved by controlling DNA repair protein levels in response to the changing DNA damage environment and involves careful synchronization of E3 ubiquitin ligases and deubiquitylation enzymes (DUBs) that control polyubiquitylation-dependent proteasomal degradation of the proteins. Given the essential role of ubiquitylation in coordinating the cellular DNA damage response, we hypothesized that DUBs will also play a central role after CDD induced by IR. However, the specific DUBs that are responsive to high-LET irradiation, which generates CDD in higher proportions compared to low-LET IR, have not been isolated to date. To this effect, we used a small interfering RNA (siRNA) screen targeting individual DUBs and analyzed cell survival in response to low- versus high-LET IR. This approach identified ubiquitin specific protease 6 (USP6), which modulates cell survival after high-LET IR by promoting efficient repair of CDD sites. We discovered that this is mediated through stabilization of poly(ADP-ribose) polymerase-1 (PARP-1) and via cell cycle progression.

## Methods and Materials

### Antibodies and siRNA

The DUB siRNA library (ON-TARGETplus) and PARP-1 siRNA containing pools of 4 individual siRNAs and an individual siRNA targeting USP6 (USP6_13 5′-CAGCUAAGAUCUCAAGUCA-3′) were from Horizon Discovery (Cambridge, UK). The non-targeting control siRNA (AllStars Negative Control siRNA) was from Qiagen (Manchester, UK). The following antibodies were used: USP6 and PARP-1 (both Santa Cruz Biotechnology, Heidelberg, Germany); phospho(T68)-Chk2 (New England Biolabs, Hitchin, UK); XRCC1 and APE1 (kindly provided by G. Dianov); γH2AX (Merck-Millipore, Watford, UK); 53BP1 (Bethyl Labs, Montgomery, AL); and actin (Sigma-Aldrich, Gillingham, UK).

### Cell culture

HeLa and UMSCC74A cells, as well as PARP-1^+/+^ and PARP-1^−/−^ mouse embryonic fibroblasts (MEFs), were cultured under standard conditions as previously described.^14^ siRNA knockdowns were performed for 48 hours using Lipofectamine RNAiMAX (Life Technologies, Paisley, UK).

### Irradiation sources

Irradiation sources are as previously described.^10^ Briefly, cells grown in 35 mm dishes were exposed to low-LET x-rays (100 kV) using CellRad x-ray irradiator (Faxitron Bioptics, Tucson, AZ). For proton irradiations, cells were irradiated directly by a ∼1 keV/μm pristine beam of 58 MeV effective energy. Alternatively, cells were irradiated using a modulator to generate a 27 mm spread-out Bragg peak and a 24.4 mm Perspex absorber to position the cells at the distal edge of the spread-out Bragg peak, corresponding to a mean proton energy of 11 MeV at a dose averaged LET of 12 keV/μm. Cells were irradiated with 3.26 MeV α-particles (LET of 121 keV/μm; dose rate of ∼1.2 Gy/min) using a ^238^Pu irradiator. Cell cycle analysis was performed by fluorescence-activated cell sorting, as also previously described.^10^

### Western blotting

Whole cell extracts were prepared, separated by sodium dodecyl sulfate polyacrylamide gel electrophoresis and analyzed by quantitative Western blotting using the Odyssey image analysis system (Li-cor Biosciences, Cambridge, UK), as previously described.^14^, ^15^

### Clonogenic assays

After irradiation in 35 mm dishes, cells were trypsinized and counted, and a defined number were seeded in triplicate into 6-well plates (250/500 for HeLa, 1000/2000 for PARP^+/+^/PARP^−/−^ MEFs, and 2000/4000 for UMSCC74A for unirradiated controls). Plating efficiencies were ∼40% for HeLa, ∼5% to 10% for PARP^+/+^/PARP^−/−^ MEFs, and ∼5% for UMSCC74A, and increasing cell numbers were used for increasing IR to account for these. Colonies were allowed to grow (∼7-10 days); they were then fixed and stained with 6% glutaraldehyde, 0.5% crystal violet for 30 minutes and counted using the GelCount colony analyzer (Oxford Optronics, Oxford, UK). Surviving fraction was expressed as colonies per treatment normalized to colonies in the untreated control from at least 3 independent experiments, apart from the DUB siRNA screen, which was from a single experiment.

### Enzyme-modified neutral comet assay

Detection of CDD using the enzyme-modified neutral comet assay was as previously described.^10^ Percent tail DNA values were calculated from at least 3 independent experiments.

### Immunofluorescent staining

Measurement of DNA repair protein foci (γH2AX and 53BP1) were examined as previously described,^14^ and mean foci/cell were calculated from at least 3 independent experiments.

## Results

### Screening for specific DUBs involved in response to high-LET IR

**Fig. 1:**
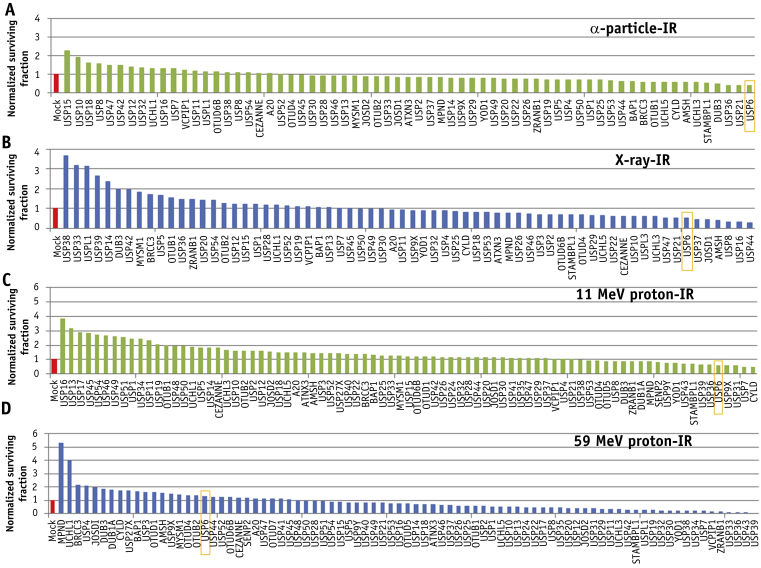
Screening of deubiquitylation enzymes involved in cell survival after high and low linear energy transfer (LET) irradiation. HeLa cells were treated with a pool of 4 small interfering RNA oligonucleotides targeting individual deubiquitylation enzymes for 48 hours and irradiated with (A) 0.5 Gy α-particles, (B) 1 Gy x-rays, (C) 2 Gy high-LET protons, or (D) 2 Gy low-LET protons. Clonogenic survival of cells was analyzed from a single experiment (using triplicate samples) and normalized against the mock treated control (red bar), which was set to 1.0 (equivalent to ∼40% cell survival after irradiation). (A color version of this figure is available at https://doi.org/10.1016/j.ijrobp.2019.02.053.)

We used an siRNA screen for up to 84 DUBs (pools of 4 siRNA duplexes) and analyzed cell survival after IR, with a focus on high-LET IR, which generates increased CDD. Using high-LET α-particle irradiation, depletion of only 4 DUBs (USP6, USP21, USP36, and DUB3) further reduced cell survival (by >50%) compared to mock transfected cells (Fig. 1A; Fig. E1A and Table E1, available online at https://doi.org/10.1016/j.ijrobp.2019.02.053), which was a different subset of DUBs to those whose depletion reduced cell survival after low-LET x-ray irradiation (Fig. 1B; Fig. E1A and Table E1, available online at https://doi.org/10.1016/j.ijrobp.2019.02.053). Using high-LET protons (Fig. 1C; Fig. E1B, available online at https://doi.org/10.1016/j.ijrobp.2019.02.053), depletion of USP6 (plus CYLD, USP7, USP31, and USP9X) also reduced cell survival by >40%. Interestingly knockdown of a large number of DUBs (27 in total) caused enhanced radiosensitivity (>50%) after low-LET protons. A complementary overexpression screen (Fig. E2A and E2B; available online at https://doi.org/10.1016/j.ijrobp.2019.02.053) revealed that overexpression of USP6 caused significantly increased cell resistance to high-LET α-particle irradiation but had no impact on low-LET x-ray irradiation.

**Fig. 2:**
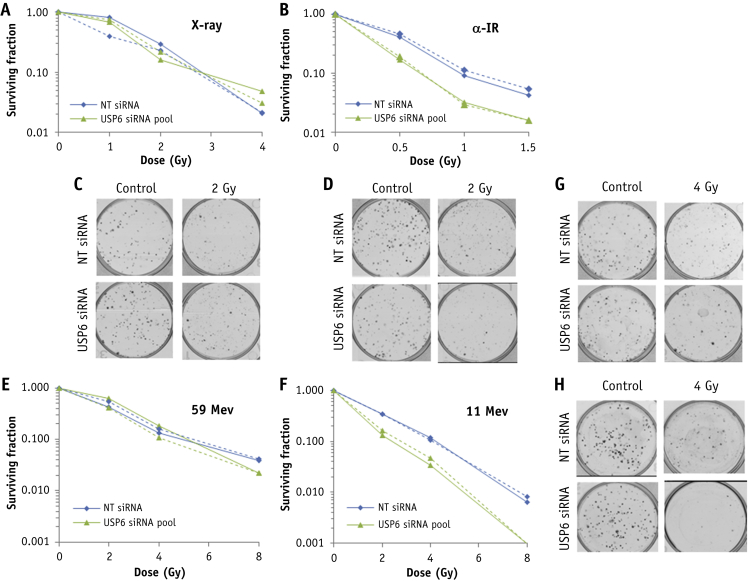
Validation of USP6 in controlling radiosensitivity in response to high linear energy transfer (LET) α-particles and protons. HeLa cells were treated with a pool of 4 small interfering RNA (siRNAs) targeting USP6 or a non-targeting control siRNA for 48 hours and irradiated with increasing doses of (A, C) x-rays, (B, D) α-particles, (E, G) low-LET protons, or (F, H) high-LET protons. Clonogenic survival of cells was analyzed from 2 independent experiments (shown by solid and dashed lines). (C, D, G, and H) Representative images of colonies in non-irradiated and irradiated plates (the latter of which were seeded with 4 times the number of cells).

**Fig. 3:**
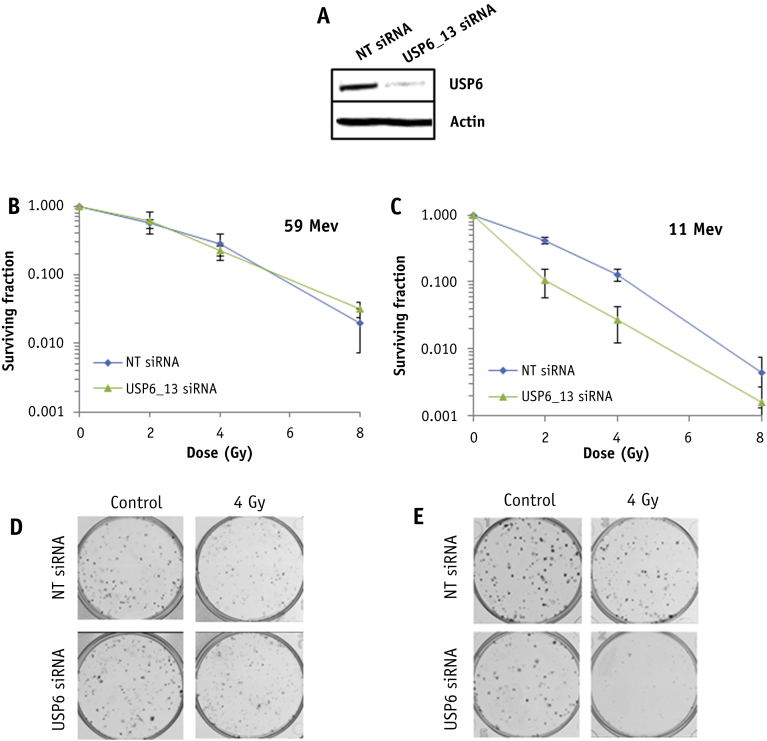
Specific targeting of USP6 leads to enhanced radiosensitivity after high linear energy transfer (LET) protons. HeLa cells were treated with an individual small interfering RNA sequence targeting USP6 (USP6_13) or a non-targeting control for 48 hours. (A) Whole cell extracts were analyzed by immunoblotting. Cells were irradiated with increasing doses of (B, D) low-LET protons or (C, E) high-LET protons. Clonogenic survival of cells was analyzed from 3 independent experiments. Shown is the mean surviving fraction ± standard error (D and E). Representative images of colonies in non-irradiated and irradiated plates (the latter of which were seeded with 4 times the number of cells).

Screening results were validated demonstrating that depletion of USP6 using a dose titration of low-LET x-rays (Fig. 2A, 2C) or low-LET protons (Fig. 2E, 2G) had no impact on the radiosensitivity of HeLa cells in comparison to non-targeting (NT) control siRNA treated cells. Note that depletion of USP6 appeared to reduce cell survival after x-rays in the siRNA screen obtained from a single experiment (Fig. 1B), which is a false positive result. In contrast, absence of USP6 caused a significant decrease in cell survival versus NT control siRNA cells after high-LET α -particle irradiation (Fig. 2B, 2D) and high-LET protons (Fig. 2F, 2H). Experiments were reproduced using a single siRNA sequence effective at suppressing USP6 protein levels (Fig. 3A) and confirmed no effect on cellular radiosensitivity in response to low-LET protons (Fig. 3B, 3D) but significantly reduced cell survival in response to high-LET protons compared with NT control siRNA cells (Fig. 3C, 3E). Depletion of USP6 was also able to specifically radiosensitive head and neck squamous cell carcinoma cells (UMSCC74A) to high-LET protons, but not low-LET protons (Fig. E3A-E3D; available online at https://doi.org/10.1016/j.ijrobp.2019.02.053).

### USP6 controls CDD repair and cell cycle progression after high-LET protons

**Fig. 4:**
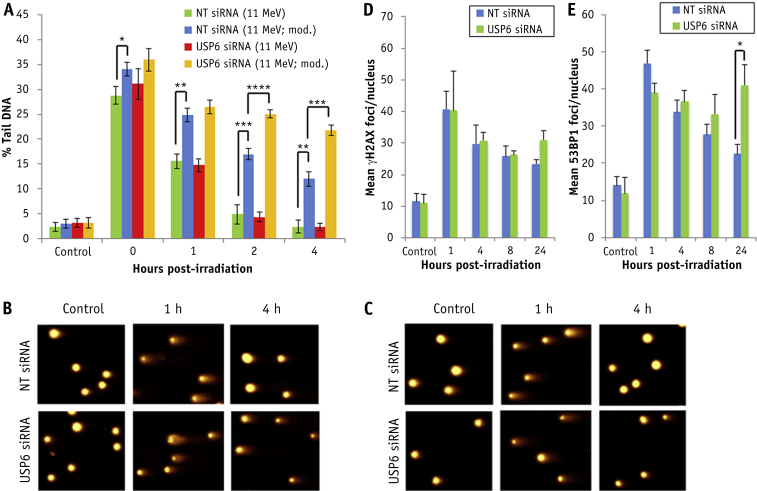
USP6 is required for efficient repair of complex DNA damage induced by high linear energy transfer (LET) protons. HeLa cells were treated with USP6 (USP6_13) small interfering RNA (siRNA) or non-targeting control siRNA for 48 hours. (A-C) Cells were irradiated with 4 Gy high-LET protons, and DNA damage was measured at various time points after ionizing radiation by using the enzyme-modified neutral comet assay after incubation in the absence (revealing double strand breaks) or presence (revealing complex DNA damage; as indicated by mod) of the recombinant enzymes APE1, NTH1, and OGG1. Shown is the mean percentage tail DNA ± standard deviation. *P < .01, **P < .005, ***P < .002, ***P < .001 as analyzed by a 1-sample *t*-test. Representative images of stained DNA in non-irradiated and irradiated cells 1 and 4 hour after irradiation in the presence (B) or absence (C) of recombinant repair enzymes. Cells were irradiated with 4 Gy high-LET protons and (D) γH2AX or (E) 53BP1 foci analyzed by immunofluorescent staining at various time points after ionizing radiation. Shown is the mean number of foci/nucleus ± standard deviation.

**Fig. 5:**
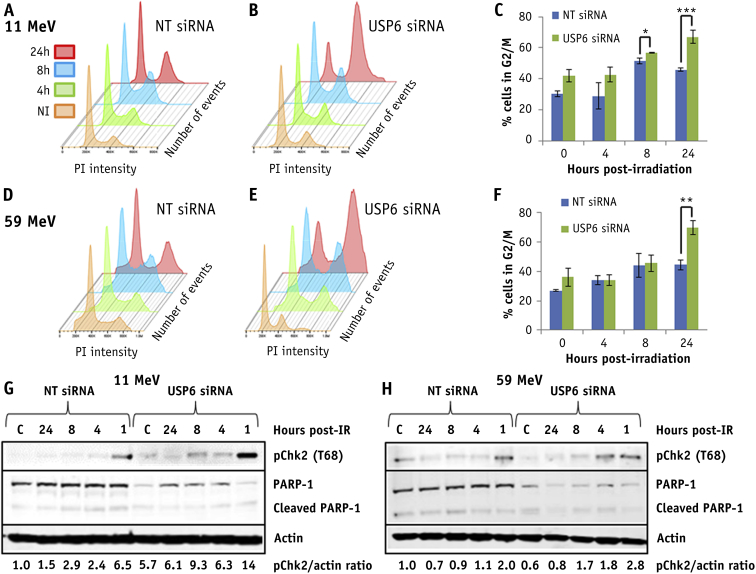
USP6 controls cell cycle progression in response to proton irradiation. HeLa cells were treated with USP6 (USP6_13) small interfering RNA (siRNA) or non-targeting control siRNA for 48 hours. At various time points after irradiation with 4 Gy (A-C) high linear energy transfer (LET) or (D-F) low-LET protons, cell cycle profiles were determined by fluorescence-activated cell sorting analysis. (C and F) Mean percentage of cells in G2/M phase ± standard error. *P < .05, **P < .02, ***P < .01 as analyzed by a 1-sample *t*-test. Cells were collected at various time points after irradiation with (G) high-LET or (H) low-LET protons, and whole cell extracts were analyzed by immunoblotting. Shown is the mean pChk2/actin ratio normalized to the levels of the untreated non-targeting control siRNA, which was set to 1.0.

Using an enzyme-modified neutral comet assay after high-LET protons, we found that depletion of USP6 significantly reduces the efficiency of CDD repair (Fig. 4A [compare blue and yellow bars] and Fig. 4B), but not DSB repair, in comparison to NT control siRNA treated cells (Fig. 4A [compare green and red bars] and Fig. 4C). Furthermore, no significant differences in γH2AX and 53BP1 foci, as surrogate markers of DSBs, were observed immediately and up to 8 hours after irradiation in cells depleted of USP6 (Fig. 4D, 4E). However, there was evidence of DSB accumulation, particularly via 53BP1 foci, 24 hours after irradiation in these cells. Cell cycle progression analysis revealed an accumulation of G2/M cells in the absence of irradiation in USP6 siRNA treated cells, which was significantly different from NT siRNA control treated cells at 8 to 24 hours after irradiation with high-LET protons (Fig. 5A-5C). Similar cell cycle profiles were observed after low-LET protons, although G2/M arrest in USP6 depleted cells was only significant at 24 hours after irradiation (Fig. 5D-5F).

In USP6 depleted cells, there was a marked increase in Chk2 phosphorylation/activation at 1 hour after high-LET protons versus NT control siRNA treated cells, which persisted at least up to 8 hours after irradiation, consistent with G2/M checkpoint activation (Fig. 5G). There was less marked Chk2 phosphorylation/activation in USP6 depleted cells after 1 hour post-irradiation with low-LET protons (Fig. 5H), suggesting less prominent activation of the G2/M checkpoint. No evidence of significantly decreased PARP-1 protein levels, and thus accumulation of cleaved PARP-1 protein as an indicator of apoptosis, in NT control or USP6 siRNA treated cells was evident after either high- or low-LET protons (Fig. 5G, 5H). However, levels of PARP-1 were noticeably lower in USP6 depleted cells.

### USP6 controls PARP-1 protein levels required for resistance to high-LET protons

**Fig. 6:**
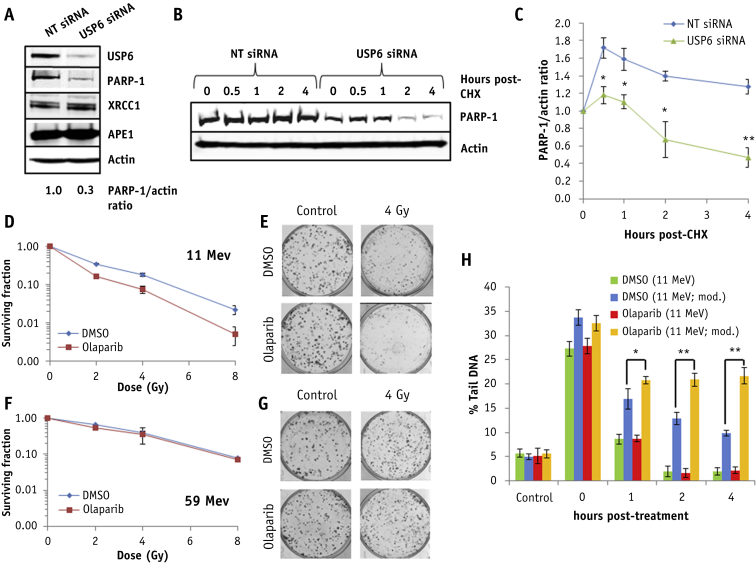
USP6 controls PARP-1 protein levels required for controlling sensitivity to high linear energy transfer (LET) protons. (A-C) HeLa cells were treated with USP6 (USP6_13) small interfering RNA (siRNA) or non-targeting control siRNA for 48 hours. (A) Whole cell extracts were analyzed by immunoblotting. (B and C) Cells were treated with cycloheximide (50 μg/mL) for the time points indicated, and whole cell extracts were analyzed by immunoblotting. (C) Mean PARP-1/actin ratio ± standard deviation. *P < .005, **P < .001 as analyzed by a 2-sample *t*-test comparing the PARP-1/actin ratio following USP6 siRNA versus non-targeting control siRNA. (D-G) HeLa cells were treated with dimethyl sulfoxide or 1 μM olaparib for 16 hours and irradiated with increasing doses of (D-E) high-LET or (F-G) low-LET protons, and clonogenic survival of cells was analyzed. Shown is the surviving fraction ± standard error. (E and G) Respective images of colonies in control and irradiated plates (the latter of which were seeded with 4 times the number of cells). (H) Cells treated with dimethyl sulfoxide or olaparib were irradiated with 4 Gy high-LET protons, and DNA damage was measured at various time points after ionizing radiation by using the enzyme-modified neutral comet assay after incubation in the absence (revealing double-strand breaks) or presence (revealing complex DNA damage; as indicated by mod) of the recombinant enzymes APE1, NTH1, and OGG1. Shown is the mean percent tail DNA ± standard deviation. *P < .01, **P < .001 as analyzed by a 1-sample *t*-test.

We substantiated significantly reduced PARP-1 protein levels (∼70%) in USP6 siRNA knockdown cells, in comparison to NT control siRNA cells (Fig. 6A). No decreases in XRCC1 and APE1 proteins, also involved in base excision/single-strand break repair, were observed in USP6 depleted cells. Cycloheximide-induced inhibition of protein synthesis revealed that PARP-1 is significantly more stable in NT control siRNA cells than in the absence of USP6 (Fig. 6B, 6C). To correlate reduced levels, and activity, of PARP-1 in USP6 depleted cells with increased sensitivity to high-LET protons, we treated cells with PARP-1 siRNA or with the PARP inhibitor olaparib. We found that both PARP-1 siRNA (Fig. E4A; available online at https://doi.org/10.1016/j.ijrobp.2019.02.053) and olaparib (Fig. 6D, 6E) led to a significant decrease in cell survival in response to high-LET protons versus NT siRNA or dimethyl sulfoxide (DMSO) treated cells, respectively, but had no impact on cell survival after low-LET protons (Fig. E4B; available online at https://doi.org/10.1016/j.ijrobp.2019.02.053) (Fig. 6F, 6G). The depletion of USP6 in combination with PARP-1 had no additive effect on reduced cell survival in response to high-LET protons, demonstrating that both proteins function within the same mechanism (Fig. E4A; available online at https://doi.org/10.1016/j.ijrobp.2019.02.053). This also had no impact on the radiosensitivity of cells after low-LET protons (Fig. E4B; available online at https://doi.org/10.1016/j.ijrobp.2019.02.053).

This evidence was strengthened by observations of increased radiosensitivity of PARP-1^−/−^ MEFs versus PARP-1^+/+^ MEFs to high-LET protons (Fig. E5A; available online at https://doi.org/10.1016/j.ijrobp.2019.02.053), but not to low-LET protons (Fig. E5B; available online at https://doi.org/10.1016/j.ijrobp.2019.02.053). Furthermore, both PARP-1 siRNA (Fig. E4C; available online at https://doi.org/10.1016/j.ijrobp.2019.02.053) and olaparib caused a significant delay in repair of CDD induced by high-LET protons that persisted for at least 4 hours after irradiation versus NT siRNA or DMSO treated cells, respectively (Fig. 6H [compare blue and yellow bars]; Fig. E6A; available online at https://doi.org/10.1016/j.ijrobp.2019.02.053), phenocopying the effect of USP6 depletion. A significant delay in the repair of CDD induced by high-LET protons was also observed in PARP-1^−/−^ MEFs, but not in PARP-1^+/+^ MEFs (Fig. E5C; available online at https://doi.org/10.1016/j.ijrobp.2019.02.053). PARP-1 depletion or PARP inhibition had no impact on the global repair of DSBs in response to high-LET protons (Fig. E4C and Fig. E6B, available online at https://doi.org/10.1016/j.ijrobp.2019.02.053; Fig. 6H [compare green and red bars]). This correlated with observations of a lack of significant differences in the kinetics of γH2AX and 53BP1 foci in cells treated with DMSO or olaparib after high-LET protons (Fig. E6C, E6D; available online at https://doi.org/10.1016/j.ijrobp.2019.02.053).

## Discussion

High-LET IR is more effective than low-LET IR at generating CDD, which, because of the presence of multiple DNA lesions, represents a significant challenge to the DNA repair machinery and is a major contributor to IR-induced cell killing. We have recently demonstrated that low-energy (relatively high-LET) protons generate increased quantities of CDD in comparison to high-energy (low-LET) protons.^10^ We also highlighted an important role for ubiquitylation in signaling and repair of CDD, mediated by the E3 ubiquitin ligases RNF20/40 and MSL2, which promote histone H2B ubiquitylation and control cell survival after irradiation with high-LET protons. We have now used an siRNA screen for DUBs to identify and characterize specific enzymes controlling cell survival after high-LET IR. We demonstrated that depletion of USP6 causes significantly increased radiosensitivity to high-LET IR (low-energy protons and α-particles), but not low-LET IR (high-energy protons or x-ray irradiation), which is mediated by instability of PARP-1 protein levels, a subsequent deficiency in CDD repair, and G2/M cell cycle checkpoint arrest. The importance of PARP-1 was corroborated by using olaparib and PARP-1 siRNA, which both mimic the effects of USP6 depletion.

Previous studies have used both siRNA screening and protein overexpression to examine the roles of DUBs in the cellular response to IR-induced DNA damage, particularly DSBs.^16^, ^17^, ^18^ These are in contrast to our study, which focused on DUBs that are responsive to high-LET IR, which generates increasing amounts of CDD. In addition, we used cell survival as an endpoint. Moreover, we have specifically examined the impact of proton irradiation on cell survival. Interestingly our siRNA screen revealed that depletion of a greater number of DUBs increased cellular radiosensitivity after low-LET protons in comparison to high-LET protons (27 and 2 DUBs, respectively, yielded a further >50% reduction in cell survival), suggesting that the cell killing effects of high-LET protons are difficult to exacerbate. In contrast, enhancing the radiosensitivity of cells after low-LET protons would appear much simpler to achieve via depletion of a number of DUBs. Another important observation is the differences in specific DUB enzymes, whose depletion leads to altered radiosensitivity in response to low-LET protons versus x-ray irradiation, although we have to be cautious in drawing conclusions from screens involving just a single experiment.

Focusing on DUBs whose depletion led to selective radiosensitization of cells after high-LET versus low-LET protons, we validated that USP6 plays a major role in this process. We are also investigating other candidates that appear to play a major role in the cellular response to high-LET radiation and are currently defining their mechanism of action. Previous reports on the cellular role and targets of USP6 are sparse, although it has been suggested to deubiquitylate Frizzled and promote Wnt signaling^19^ and to deubiquitylate Jak1, causing activation of the STAT3 signaling pathway.^20^ USP6 has also been demonstrated to regulate the stability of the c-Jun transcription factor,^21^ and high USP6 protein expression has been observed in bone and soft-tissue tumours.^22^ We now extend these observations by demonstrating that USP6 plays a critical cellular role in controlling radiosensitivity to high-LET IR and in the cellular DNA damage response.

Indeed, in USP6 depleted cells we observed reduced efficiency of CDD repair as a consequence of a significant reduction in PARP-1 protein levels and stability and a G2/M cell cycle arrest. Importantly we showed that cells treated with the PARP inhibitor olaparib or with PARP-1 siRNA, along with PARP^−/−^ MEFs, also displayed increased radiosensitivity to high-LET protons as a consequence of deficiencies in CDD repair. No decrease in cell survival was observed in HeLa cells with olaparib or with PARP-1 siRNA, or in PARP^−/−^ MEFs, in combination with low-LET protons, although this phenotype will not likely be shared across all cell lines. Nevertheless, this would suggest synergy between PARP inhibition (or a deficiency in PARP-1 protein) and CDD induced by high-LET IR in promoting cancer cell killing, similar to the concept of synthetic lethality observed with PARP inhibitors in combination with BRCA (DSB repair)-deficient cancers.^23^, ^24^ These data also correlate with our previous evidence demonstrating that CDD induced by high-LET protons appears to be largely single-strand break in nature and thus dependent on PARP-1, and predictably other single-strand break repair proteins, for repair.^10^ However, we now advance these findings and demonstrate here that both PARP-1 protein and activity are required for CDD repair and in promoting cell survival.

Although PARP-1 is known to be regulated by ubiquitylation,^12^ particularly the poly(ADP-ribosyl)ated form of the protein by the E3 ubiquitin ligases Iduna/RNF146^25^ and CHFR,^26^ to our knowledge the identification of a specific DUB involved in controlling PARP-1 protein levels has not previously been reported. Therefore, our data would suggest an interplay between USP6 and Iduna and/or CHFR in the controlled regulation of PARP-1 protein levels, which requires further clarification. Nevertheless, our evidence now indicates that USP6 and PARP-1 should be considered important factors in the responsiveness of cancer cells to radiation therapy, particularly proton beam therapy that can generate high-LET protons and CDD at the distal end of the Bragg peak.

## Supplementary Data

Table E1 and Figures E1-E6
