# Supplementary material for: Characterisation of Deubiquitylating Enzymes in the Cellular Response to High-LET Ionizing Radiation and Complex DNA Damage
Source: Int J Radiat Oncol Biol Phys. 2019 Jul 1;104(3):656–65. doi: 10.1016/j.ijrobp.2019.02.053 (PMC6542414; doi:10.1016/j.ijrobp.2019.02.053)
Supplement: Table E1 and Figures E1-E6 [file mmc1.docx]

**Supplementary Table**

**Table E1.** List of DUBs whose depletion leads to enhanced or reduced sensitivity to x-ray, α-particle or low/high energy proton irradiation. The proteins listed below are derived from an siRNA screen utilising clonogenic assay survival data that demonstrate a >50 % change in cell survival relative to mock treated cells following a single dose of radiation (USP6 is indicated in brackets in sensitising cells to high-LET protons through a 40 % decrease in cell survival post-irradiation). Depletion of only UCHL1 (highlighted in red) and USP7 (highlighted in blue) caused significant cellular resistance and sensitivity, respectively to both high and low-LET protons.

**
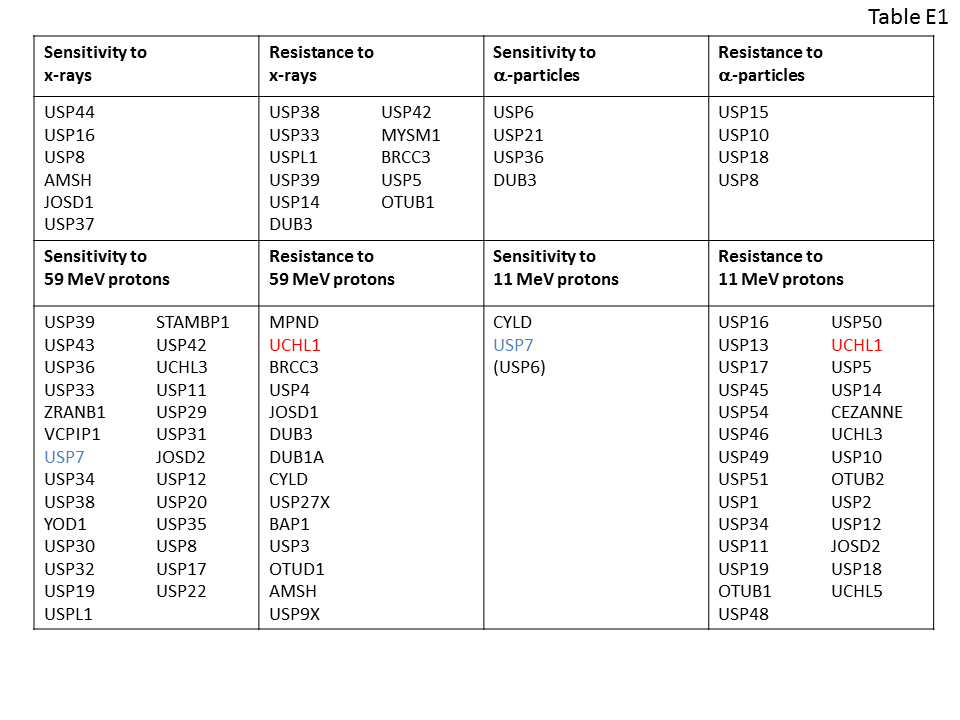
**

**Supplementary Figures**

**
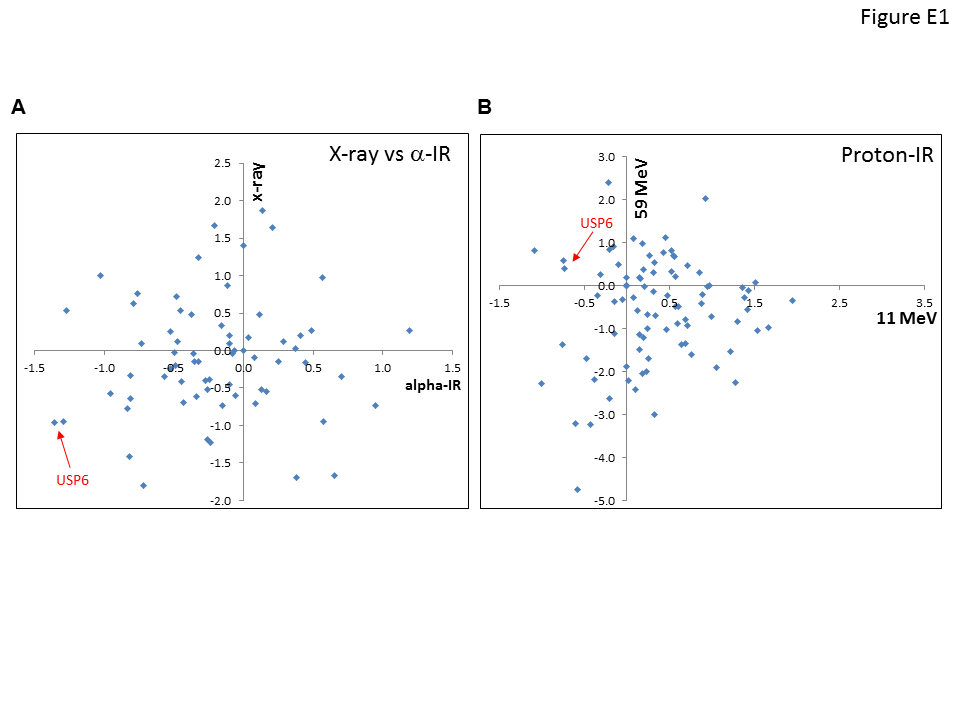
**

**Figure E1.** Comparison of cell survival in the absence of DUBs following high- versus low-LET irradiation. HeLa cells were treated with a pool of four siRNAs targeting individual DUBs for 48 h, and irradiated with either (A) 0.5 Gy α-particles versus 1 Gy x-rays, or (B) 2 Gy high-LET protons versus low-LET protons. Clonogenic survival was analysed from a single experiment (using triplicate samples) and normalised against the mock treated control (red bar) which was set to 1.0 (equivalent to ~40 % cell survival post-irradiation). Log2 plots of the data are shown demonstrating fold changes in cell survival using the respective low-LET radiation (y-axis) versus high-LET radiation (x-axis).

**
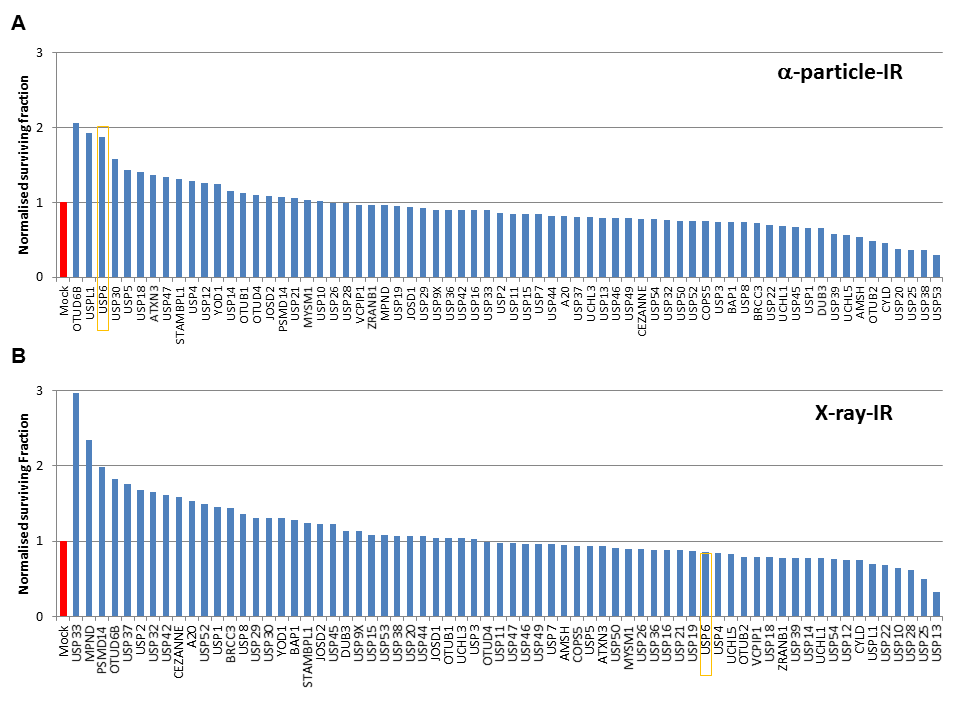
**

**Figure E2.** Overexpression screen of DUBs involved in the survival of cells following α-particle and x-ray irradiation. HeLa cells in 35 mm dishes were treated with 750 ng of mammalian expression plasmids for individual DUBs for 24 h, and irradiated with (A) 0.5 Gy α-particles or (B) 1 Gy x-rays. Clonogenic survival of cells was analysed from a single experiment (using triplicate samples) and normalised against the mock treated control (red bar) which was set to 1.0 (equivalent to ~40 % cell survival post-irradiation).

**
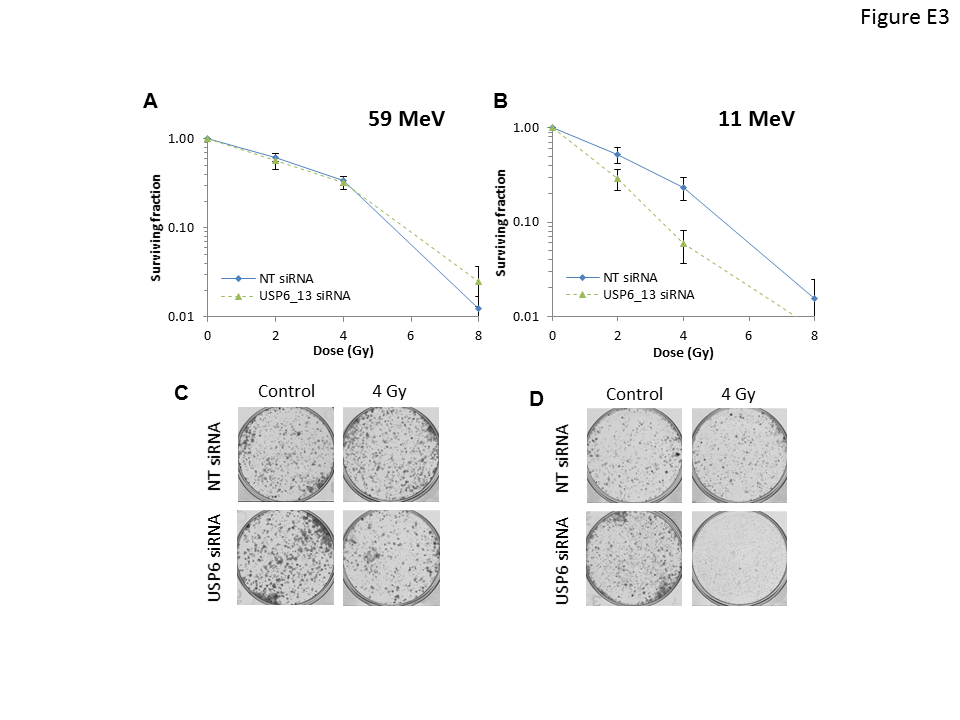
**

**Figure E3.** Specific targeting of USP6 leads to enhanced radiosensitivity of head and neck squamous cell carcinoma cells to high-LET protons. UMSCC74 cells were treated with an individual siRNA targeting USP6 (USP6_13) or a non-targeting (NT) control for 48 h. Cells were irradiated with increasing doses of (A) low-LET protons or (B) high-LET protons. Clonogenic survival of cells was analysed from three independent experiments and shown is mean surviving fraction±S.E. (C, D) Respective images of colonies in control and irradiated plates (the latter of which were seeded with four times the number of cells).

**
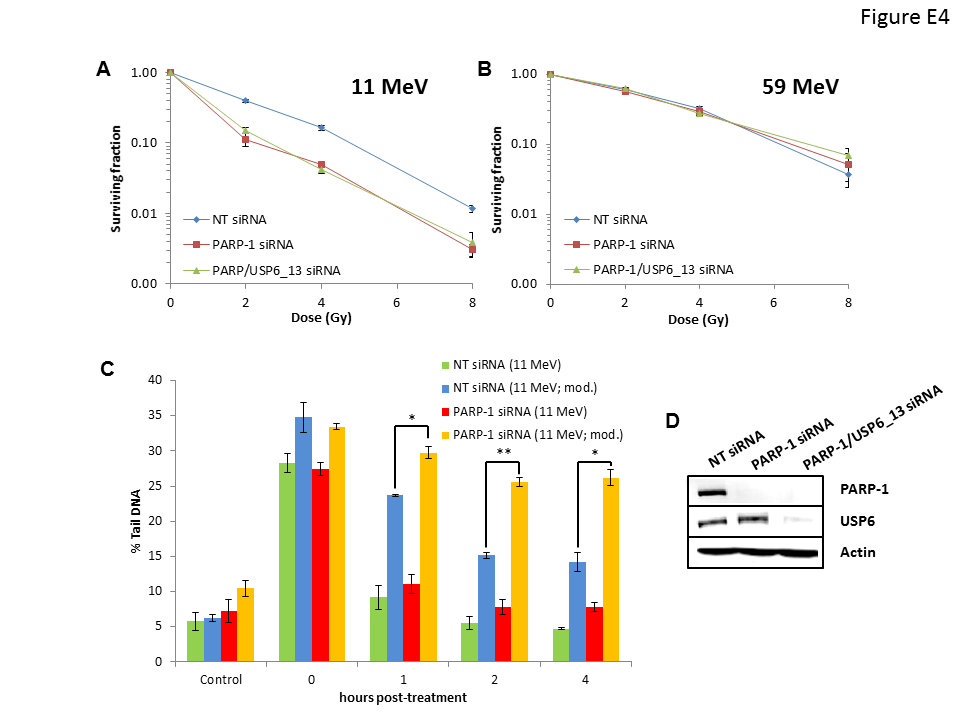
**

**Figure E4.** PARP-1 protein is required for controlling cellular sensitivity to high-LET protons by enhancing CDD repair. (A-D) HeLa cells were treated with PARP-1 siRNA alone or in combination with USP6 (USP6_13) siRNA, or with a non-targeting (NT) control siRNA for 48 h. Cells were irradiated with increasing doses of (A) high-LET or (B) low-LET protons and clonogenic survival of cells analysed. Shown is the surviving fraction±S.E. (C) Alternatively cells were irradiated with 4 Gy high-LET protons and DNA damage measured at various time points post-IR by the enzyme modified neutral comet assay following incubation in the absence (revealing DSBs) or presence (revealing CDD; as indicated by mod) of the recombinant enzymes APE1, NTH1 and OGG1. Shown is the mean % tail DNA±S.D. *p<0.01, **p<0.005 as analysed by a one sample *t*-test. (D) Whole cell extracts were analysed by immunoblotting using the indicated antibodies.

**
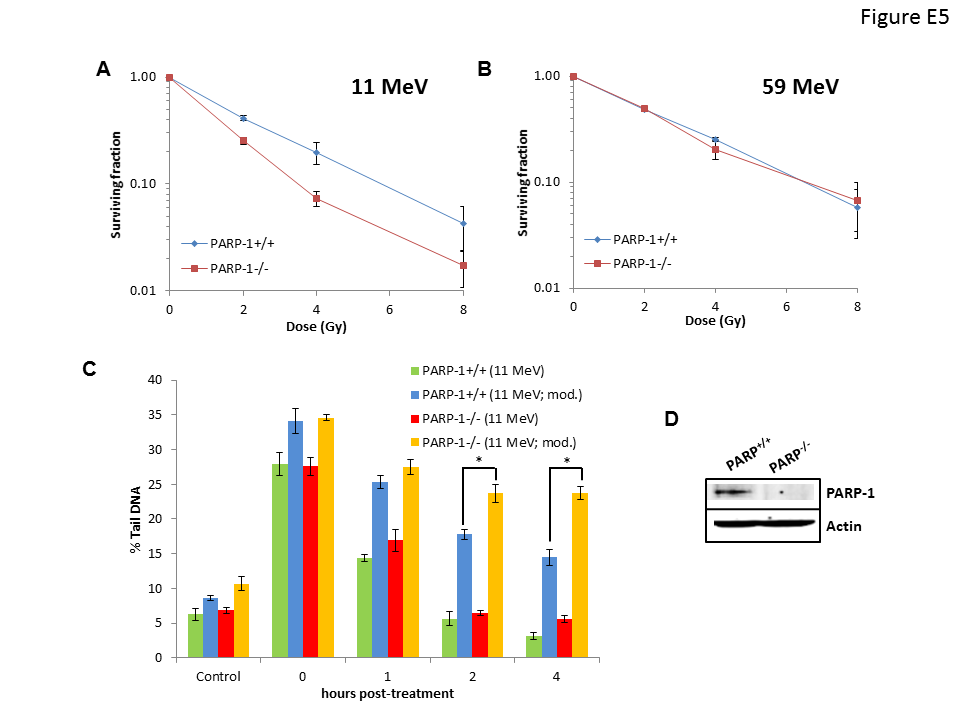
**

**Figure E5.** PARP-1^-/-^ MEFs display increased radiosensitivity to high-LET protons through deficiencies in CDD repair. (A-D) PARP-1^+/+^ and PARP-1^-/-^ MEFs were irradiated with increasing doses of (A) high-LET or (B) low-LET protons and clonogenic survival of cells analysed. Shown is the surviving fraction±S.E. (C) Alternatively cells were irradiated with 4 Gy high-LET protons and DNA damage measured at various time points post-IR by the enzyme modified neutral comet assay following incubation in the absence (revealing DSBs) or presence (revealing CDD; as indicated by mod) of the recombinant enzymes APE1, NTH1 and OGG1. Shown is the mean % tail DNA±S.D. *p<0.01 as analysed by a one sample *t*-test. (D) Whole cell extracts were analysed by immunoblotting using the indicated antibodies.

**
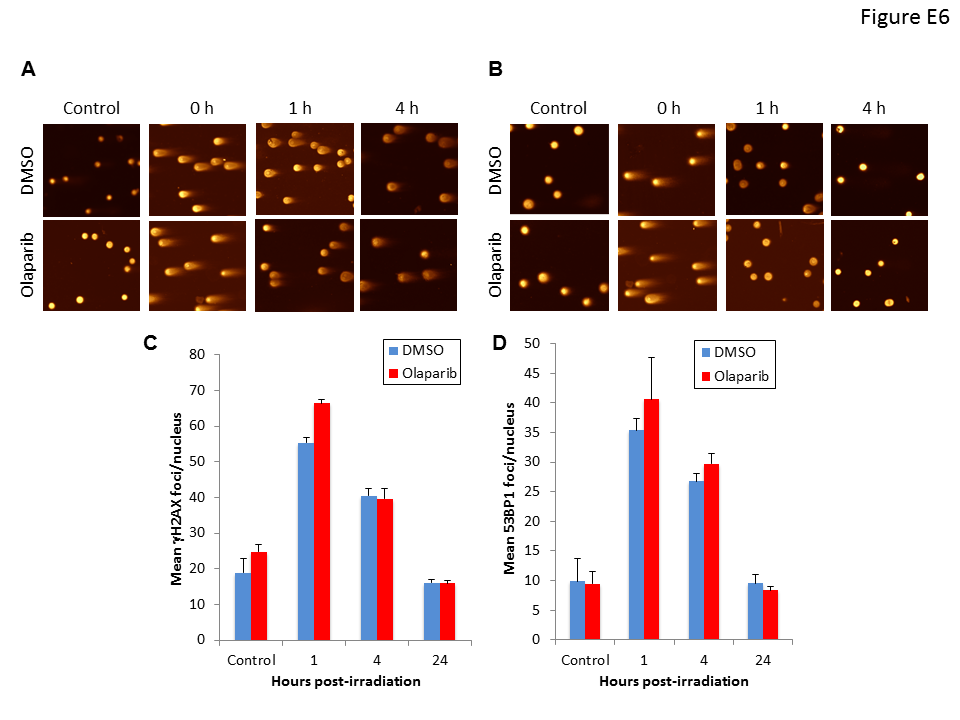
**

**Figure E6.** Inhibition of PARP using olaparib causes persistent CDD formation following high-LET protons. (A-B) HeLa cells were treated with DMSO or olaparib (0.1 µM) for 16 h, cells were irradiated with 4 Gy high-LET protons and DNA damage measured at various time points post-IR by the enzyme modified neutral comet assay following incubation in the presence (A; revealing CDD; as indicated by mod) or absence (A; revealing DSBs) of the recombinant enzymes APE1, NTH1 and OGG1. Shown are respective images of stained DNA in control and irradiated cells immediately and 1 or 4 h post-irradiation. Alternatively cells following DMSO or olaparib treatment were irradiated with 4 Gy high-LET protons and (C) γH2AX or (D) 53BP1 foci analysed by immunofluorescent staining at various time points post-IR. Shown is the mean number of foci/nucleus±S.D.
